# Highly pathogenic avian influenza H5 clade 2.3.4.4 and human new pandemic H1N1 virus exposure in domestic cats with outdoor access in the Netherlands in 2024

**DOI:** 10.1177/1098612X261461681

**Published:** 2026-06-09

**Authors:** Mirjam BHM Duijvestijn, Els M Broens, Nancy NMP Schuurman, Johannes CM Vernooij, Judith MA van den Brand, Jaap A Wagenaar, Frank JM van Kuppeveld, Josanne H Verhagen, Cornelis AM de Haan

**Affiliations:** 1 Division of Infectious Diseases and Immunology, Department of Biomolecular Health Sciences, Faculty of Veterinary Medicine, Utrecht University, The Netherlands; 2 Division of Farm Animal Health, Department of Population Health Sciences, Faculty of Veterinary Medicine, Utrecht University, The Netherlands; 3 Division of Pathology, Department of Biomolecular Health Sciences, Faculty of Veterinary Medicine, Utrecht University, The Netherlands

**Keywords:** Avian influenza, influenza A virus, pandemic preparedness, serosurveillance, zoonosis

## Abstract

**Objectives:**

Cats are susceptible to highly pathogenic avian influenza H5 clade 2.3.4.4 (HPAI H5) and human new pandemic H1N1 (H1N1_pdm09_) influenza A viruses. A simultaneous infection with multiple influenza A virus subtypes could potentially result in the generation of reassortant viruses with enhanced zoonotic potential. Previously, high seropositivity (11.8%) to HPAI H5 virus has been detected in rural stray cats in the Netherlands, presumably through contact with or feeding on infected birds. Seropositivity was much lower (0.46%) in concurrently sampled domestic cats with unknown outdoor access, which were frequently (4.6%) seropositive to H1N1_pdm09_ virus. As outdoor access is expected to increase the risk of HPAI H5 exposure, in this study we determined seropositivity to HPAI H5 as well as H1N1_pdm09_ in domestic cats with known outdoor access.

**Methods:**

In 2024, sera from 254 outdoor cats were collected and screened for antibodies to HPAI H5 and H1N1_pdm09_ virus using in-house developed ELISAs and haemagglutination inhibition assays (HAIs).

**Results:**

Antibodies to HPAI H5 virus were detected in seven (2.8%) cat sera (95% confidence interval [CI] 1.1–5.6) by ELISA, but not by HAI. Antibodies to H1N1_pdm09_ were detected in 14 (5.5%) sera (95% CI 3.1–9.1), of which seven (2.8%) were positive by HAI (95% CI 1.1–5.6). Two sera (0.79%) reacted in ELISA to both HPAI H5 and H1N1_pdm09_ (95% CI 0.1–2.8).

**Conclusions and relevance:**

Antibodies to both HPAI H5 and H1N1_pdm09_ viruses were detected in outdoor domestic cats, with a higher seroprevalence for H1N1_pdm09_. Seropositivity for HPAI H5 was higher than was previously detected in domestic cats with unknown outdoor access, but lower than in stray cats. This warrants further investigation into the potential role of outdoor domestic cats as mixing vessels and as a source of (novel) zoonotic viruses.

## Introduction

Highly pathogenic avian influenza H5 clade 2.3.4.4 viruses (referred to as HPAI H5) have been enzootic among wild birds worldwide, including the Netherlands, since 2020.^1–4^ These avian-origin influenza A viruses (IAVs) have spilled over to carnivores,^5–7^ including domestic cats.^8–14^ In cats, HPAI H5 virus exposure may have occurred through direct and/or indirect (ie, contaminated faeces or feathers^15^) contact with infected birds,^8,13,16–19^ but also through consumption of contaminated raw meat or petfood^10,12,20,21^ or, in the USA, contaminated raw milk.^22,23^ The increasing number of HPAI H5-infected cats in the USA since 2022,^24^ where cat-to-cat,^25^ cat-to-human^26^ and human-to-cat^27^ transmission may have occurred, emphasises the importance of cats as hosts and sentinels of HPAI H5 viruses. Domestic cats can also be infected with currently circulating human pandemic H1N1 (H1N1_pdm09_) IAVs.^28–32^

Although experimental HPAI H5 virus infection of cats resulted in clinical signs and death,^33–36^ the detection of antibodies against HPAI H5 and H1N1_pdm09_ in apparently healthy domestic cats indicated that exposure of cats to IAVs may remain clinically unnoticed.^11,14,16,18,37^ Cat-to-cat transmission of both these viruses has been experimentally demonstrated,^31,33,35^ while transmission was suspected but not confirmed under natural conditions.^27^ (Co)infection of cats with HPAI H5 and H1N1_pdm09_ viruses might result in the generation of novel viruses with zoonotic potential, through reassortment and/or adaptation. Reassorted viruses containing segments from different avian viruses have been isolated from cats,^38–40^ including a triple reassortant H3N8 IAV that was simultaneously detected in a child from the same household, suggesting cross-species zoonotic transmission.^39^

Previous studies in the Netherlands showed that shelter cats (consisting of 86.4% ex-stray cats) sampled in 2016, and rural living stray cats (either owned cats gone astray, community cats or feral cats, as described previously^41^) sampled between 2020 and 2023, were more frequently HPAI H5 seropositive (7.3% and 11.8%, respectively) than domestic (owned) cats with unknown outdoor access sampled in 2019 or between 2020 and 2023 (1.5% and 0.46%, respectively).^11,37^ Antibodies to H1N1_pdm09_ virus were common (15.3% and 4.6%, respectively) in domestic cat cohorts.^11,37^ Of note, in these studies, ELISAs based on recombinant hemagglutinin (HA) protein were used, which were shown to be more sensitive for detection IAV antibodies in cat sera than an ELISA based on the IAV nucleoprotein^37^ or than haemagglutination inhibition assays (HAIs),^11,37^ in agreement with other studies.^42,43^

Based on their lifestyle, domestic cats with outdoor access may be at higher risk for exposure to HPAI H5 through infected wild birds than indoor cats, while they are also at risk for H1N1_pdm09_ through infected caretakers.^11,28,37^ Outdoor domestic cats may therefore not only be a source of HPAI H5 (or H1N1_pdm09_) viruses to humans, but potentially also of novel zoonotic viruses resulting from reassortment after coinfection. The aim of the current study was to gain more insight into the exposure of domestic cats with known outdoor access in the Netherlands to both these viruses. We therefore determined the presence of HPAI H5 and H1N1_pdm09_ antibodies and compared the seroreactivity of the cohort sampled in this study with that of domestic cats and stray cats sampled previously, which we hypothesise to have lower and higher seroreactivity to HPAI H5, respectively.

## Materials and methods

### Sample collection

After obtaining owner-informed consent, 24 veterinary practices submitted surplus serum samples from domestic cats. The large majority of these samples (n = 254) were from cats with outdoor access (outdoor cats), while a limited number (n = 33) came from indoor cats. None of the cats sampled were fed raw meat. We initially planned to only include outdoor cats in the study; however, during the sampling phase we received indoor cat samples, so we included this indoor cat cohort as a control group in the study (Table 1). Outdoor cats had access to a garden or non-confined outdoor area. Indoor cats lived strictly indoors, or had access to an outdoor enclosure or balcony, but were restricted in direct bird contact. Data on cats’ age, sex and postal code of the home address accompanied the samples.

**Table 1:** Sample description of collected domestic cat sera

Description	Outdoor cats (n = 254)	Indoor cats (n = 33)
Age (years)	10.7 (12.0 [7.0–14.0])	8.9 (10.0 [3.0–14.0])
Male/female	149/105	10/23

Data are mean (median [interquartile range]) unless otherwise indicated

### Antibody detection by ELISA and HAI

All cat sera were analysed simultaneously in three in-house developed ELISAs, as described previously,^11^ based on the HA protein of HPAI H5N8 clade 2.3.4.4 virus (A/Chicken/NL/14015526/2014,^11,37^ referred to as HPAI H5), low pathogenic avian influenza (LPAI) H5N2 virus (A/Common Teal/NL/4/2022,^11^ referred to as LPAI H5) and human H1N1_pdm09_ IAV (A/California/04/2009, referred to as H1).^11,37^ The ELISA cut-off (five times the optical density [OD] value measured at 450 nm of a negative specific pathogen free cat serum) was calculated per assay, and the ELISA results were depicted as OD ratios (OD value:cut-off). Specific binding of a single serum sample that reacted positively in all three ELISAs was confirmed by lack of binding above the cut-off to an uncoated but blocked well. Sera positive in at least one of the ELISAs, and 24 randomly selected ELISA-negative sera, were analysed in HAIs. In the HAIs, the same HA proteins used in the ELISAs were coupled to mi3 nanoparticles, as described previously.^11,37^

### Data analysis

Data analysis and visualisation was conducted using SPSS version 28.0 (IBM), GraphPadPrism version 10 (GraphPad Software) and Datawrapper (https://www.datawrapper.de). To calculate seroproportions with confidence intervals (CIs), the exact binominal test was used. The association of age and sex with HPAI H5 or H1 seropositivity was explored in a univariable analysis using the χ^2^ or Fisher’s exact test (when expected count <5), and odds ratios with 95% CIs were calculated. A *P* value <0.05 was considered statistically significant. The indoor cat cohort was excluded from detailed statistical analyses owing to the low sample size.

### Reuse of data

The seroreactivity and seroprevalence of the samples analysed in this study were compared with those of cohorts of domestic cats with unknown outdoor access and stray cats obtained previously (2020–2023) by us and that were analysed using the same HPAI H5 and H1 proteins and ELISA and HAI methodology as in the current study.^11^ Global Initiative on Sharing All Influenza Data (GISAID) EpiFLu (https://gisaid.org, accessed on 5 March 2025) and European Food Safety Authority (EFSA; https://hpai.efsa.aus.vet, accessed on 12 December 2024) databases were used to obtain data on the presence of HPAI clade 2.3.4.4-infected wild birds in the Netherlands.^44^ All data were reused with permission when applicable.

## Results

### Sample collection

**Figure 1:**
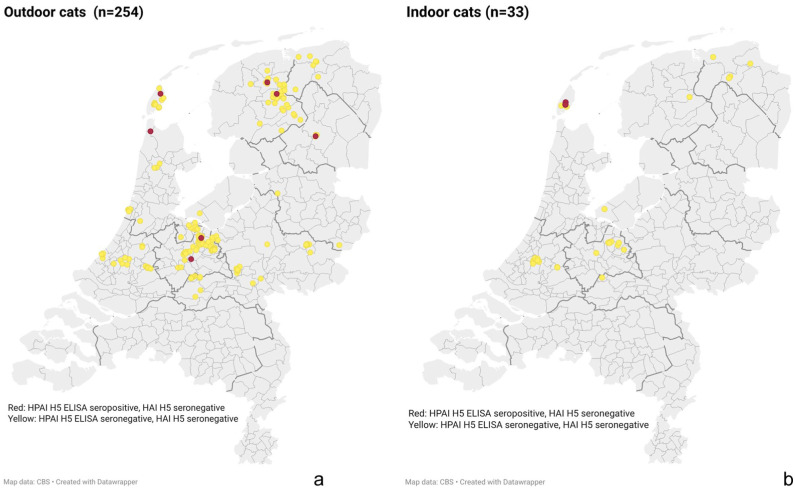
Geographic distribution of sampled domestic cats in the Netherlands, 2024: (a) 253/254 sampled outdoor cats and (b) 33 indoor cats. Circles correspond to home addresses of individual cat owners based on the four digits of the postal code. Red and yellow circles indicate positive and negative reactivity in the highly pathogenic avian influenza (HPAI) H5 ELISA, respectively. Municipalities and provinces are indicated by light and dark grey lines respectively (www.cbs.nl)

Sera from 254 outdoor cats and 33 indoor cats (total n = 287 cats) were collected between January and September 2024 (mean number of samples per veterinary practice 11; median 17 [range 2–55]). Targeted sampling was performed for areas where previously HPAI H5 seropositive stray cats had been detected,^11^ resulting in clustering of samples in the north and centre of the Netherlands (Figure 1a,b).

### Seroreactivity

**Figure 2:**
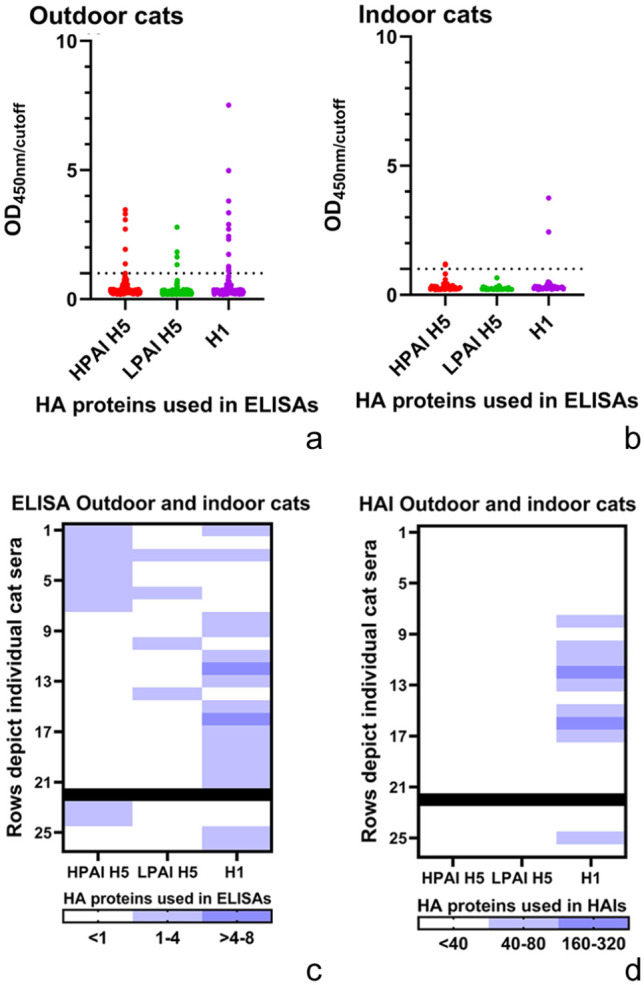
Reactivity in cat sera to influenza A viruses. Reactivity of serum samples from (a) 254 outdoor cats and (b) 33 indoor cats in ELISAs depicted as dot plots. The dotted line indicates the cut-off (ELISA optical density [OD] ratio = 1). Heatmaps of (c) ELISA and (d) haemagglutination inhibition assay (HAI) reactivity from 21 outdoor cats and four indoor cats positive for antibodies to highly pathogenic avian influenza (HPAI) H5 and/or low pathogenic avian influenza (LPAI) H5 and/or H1 in ELISA. The outdoor and indoor cat samples are presented above and below the black horizontal bar, respectively. The blue colour intensity corresponds with ELISA or HAI-binding reactivity of the sera based on OD ratio or HAI titre as indicated. The white colour depicts reactivity below the cut-off. HA = haemagglutinin protein

Seropositivity to HPAI H5 in ELISA was detected in 7/254 (2.8%) outdoor cat sera (95% CI 1.1–5.6) (Figure 2a,c) but not in HPAI H5-HAI (Figure 2d). Reactivity to LPAI H5 in ELISA was detected in 4/254 (1.6%) sera (95% CI 0.43–4.0), but not in LPAI H5-HAI. Reactivity to H1 in ELISA was detected in 14/254 (5.5%) sera (95% CI 3.1–9.1), of which seven (50.0%) were positive in H1-HAI (2.8%; 95% CI 1.1–5.6). Two outdoor cat sera reacted positively to both HPAI H5 and H1 in ELISA, with similar OD ratios (0.79%, 95% CI 0.1–2.8). Of 33 indoor cat sera, two (6.1%; 95% CI 0.74–20.2) displayed low reactivity to the HPAI H5 ELISA (Figure 2b) but not in HPAI H5-HAI. Two other indoor cat sera (6.1%; 95% CI 0.74–20.2) reacted positive in ELISA to H1, one of which was also positive in the H1-HAI (Figure 2d).

**Figure 3:**
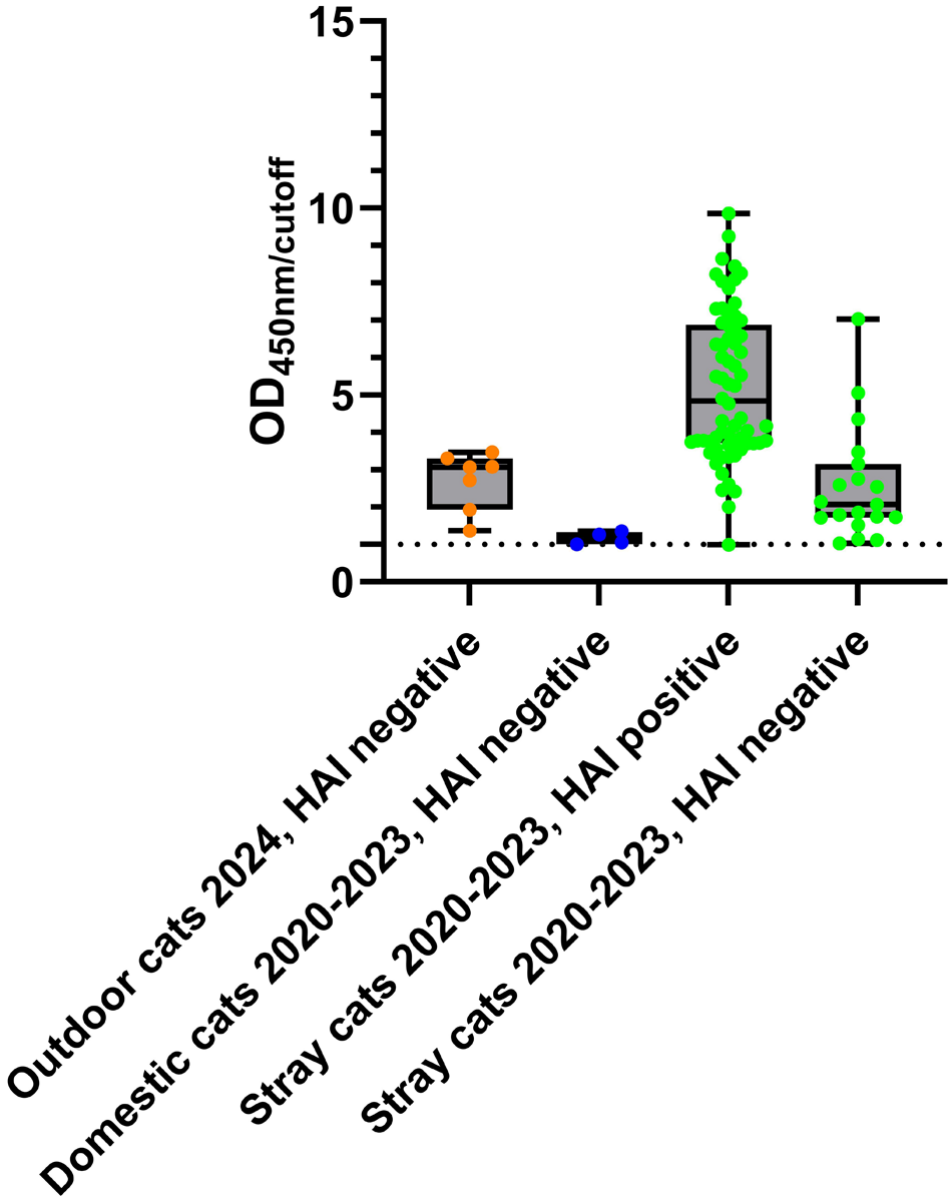
Seroreactivity analysis of highly pathogenic avian influenza H5 ELISA-seropositive and haemagglutination inhibition assay (HAI)-negative cats. Optical density (OD) ratios of ELISA-positive, HAI-negative outdoor domestic cat sera (n = 7) were plotted. For comparison, similar ratios from ELISA-positive, HAI-negative (n = 4) domestic cat sera, ELISA-positive and HAI-positive (n = 66) and ELISA-positive, HAI-negative (n = 19) stray cat sera obtained using similar methods^11^ are also plotted. The reactivity is depicted as dot plots and boxplots. The HAI cut-off was set at titre 40

In our previous study,^11^ although 79.3% of HPAI H5 ELISA positive stray cat sera were positive in HAI, none of the HPAI H5 ELISA positive cat sera in this study were positive in HAI. We therefore analysed the degree of seroreactivity of seropositive outdoor cats from this study and compared this with that of stray cats and domestic cats analysed previously using the same assays^11^ and that were either HAI positive or negative (Figure 3). The range of HPAI H5 ELISA OD ratios of the positive outdoor cat sera observed in this study was comparable to ELISA-positive but HAI-negative stray cat sera, and was lower than stray cat sera that were both ELISA and HAI positive, and higher than domestic cat sera that were ELISA positive and HAI negative (Figure 3). We conclude that the absence of detectable HAI titres in the outdoor cat sera in this study can be explained by low antibody levels combined with a lower sensitivity of the HAI compared with the ELISA.^11,37^

### Age and sex do not associate with HPAI H5 or H1 ELISA seropositivity in outdoor cats

Previously, we found that HPAI H5 seropositivity was significantly higher in stray cats aged 3 years or older compared with younger cats.^11^ Age and sex were, however, not significantly associated with HPAI H5 or H1 ELISA seropositivity in the outdoor domestic cats analysed in this study (Table 2).

**Table 2:** Associations of age and sex with seropositivity to highly pathogenic avian influenza (HPAI) H5 virus or human H1 virus in outdoor cats

Characteristics	Cats sampled	HPAI H5 ELISA seropositive	OR (95% CI)	*P* value
HPAI H5 virus				
Age (years)*				
0<3	34 (13.4)	1 (2.9)	Ref	
⩾3	219 (86.6)	6 (2.7)	0.93 (0.11–8.0)	0.95
Sex				
Female	105 (41.3)	3 (2.9)	Ref	
Male	149 (58.7)	4 (2.7)	0.94 (0.21–4.3)	0.93
Characteristics	Cats sampled	H1 ELISA seropositive	OR (95% CI)	*P* value
Human H1 virus				
Age (years)*				
0<3	34 (13.4)	0 (0)	Ref	
⩾3	219 (86.6)	14 (6.4)	4.9^†^ (0.12–34.1)	0.23
Sex				
Female	105 (41.3)	5 (4.8)	Ref	
Male	149 (58.7)	9 (6.0)	1.3 (0.42–4.0)	0.66

Data are n (%) unless otherwise indicated

* Age was unavailable for one cat

† Firth correction applied to correct for categories with zero events

CI = confidence interval; OR = odds ratio; Ref = reference category

## Discussion

We analysed HPAI H5 and H1N1_pdm09_ ELISA seropositivity in domestic cats with outdoor access. The proportion of HPAI H5-seropositive outdoor cats (2.8%) found in this study was higher than of domestic cats with unknown outdoor access (0.46%; but significance was not reached) and significantly lower than of stray cats (11.8%), sampled previously (2020–2023) by us in the Netherlands, and subjected to a similar serological analysis^11^ (Table 3), which is in agreement with our hypothesis. The indoor cat cohort was excluded from this comparison because of the low sample size. A comparable HPAI H5 seropositivity (2.6%) was observed in a French cat cohort consisting of 642 (2.2%) outdoor cats and 86 (5.8%) stray cats sampled in 2023–2025.^14^

**Table 3:** Comparison of influenza A virus ELISA seropositivity in different cat cohorts in the Netherlands

ELISA antigen	Outdoor cats current study (1/2024–9/2024) (n = 254)	Domestic* cats sampled previously (11/2020–3/2023) (n = 871)^11^	Stray cats sampled previously (10/2020–6/2023) (n = 701)^11^
HPAI H5 – positive	7 (2.8)	4 (0.46)	83 (11.8)
	95% CI: 1.1–5.6	95% CI: 0.13–1.2	95% CI: 9.5–14.5
H1 – positive	14 (5.5)	40 (4.6)	35 (5.0)
	95% CI: 3.1–9.1	95% CI: 3.3–6.2	95% CI; 3.5–6.9^†^

Data are n (%) unless otherwise indicated

* Domestic cats with unknown outdoor access

† Cross-reactivity of highly pathogenic avian influenza (HPAI) H5 antibodies to H1 in ELISA was suspected in 30/35 samples.^11^ H1N1_pdm09_ seropositivity found in stray cats may be an overestimation resulting from cross-reactivity between HPAI H5 and H1 in ELISA, as most of these cats displayed low reactivity to H1 (optical density [OD] ratio <4) and high reactivity to HPAI H5 (OD ratio >4),^11^ which was not observed for the domestic cat cohorts analysed previously^11^ and in this study

CI = confidence interval

Outdoor access is a presumed important prerequisite of HPAI H5 virus exposure.^11,14,37^ Direct or indirect contact with infected birds is more likely for permanent outdoor living stray cats than for outdoor domestic cats that spend part of their life indoors. Moreover, in contrast to domestic cats,^45^ stray cats need to prey or scavenge on birds to survive,^46,47^ which adds to their exposure. In agreement herewith, absence of hunting behaviour was shown to be a significant protective factor.^14^ In addition, whereas stray cats may scavenge on dead waterfowl that are highly susceptible to HPAI H5 virus,^1,3,4,46^ outdoor cats mostly prey on smaller songbirds or pigeons,^48^ in which HPAI H5 virus infections were less frequently detected in the Netherlands.^4^ The absence of a significant association of age with HPAI H5 seroprevalence in this study may have been due to the low number of HPAI H5 seropositive cats and lower sample size compared with the previous study (11). The lower HPAI H5 ELISA seropositivity in domestic cats with unknown outdoor access sampled previously^11^ may be due to sampling a substantial proportion of strictly indoor cats (~40% cats in the Netherlands live strictly indoors).^11,37,49^ As secondary data, in this study we also found HPAI H5 antibodies in two indoor cats. These cats originate from the island Texel where very high numbers (25.9%) of HPAI H5 seropositive stray cats were reported previously.^11^ One of these cats had limited outdoor access via an enclosure and may have been indirectly exposed.^15^ The other cat was reported to strictly live indoors. However, historical information on the living condition of these two cats is unavailable, limiting further interpretation of these positive results. To conclude, frequency of (in)direct bird contact, contact with high-at-risk bird species and scavenging may partly explain differences in seropositivity among cat cohorts.

**Figure 4:**
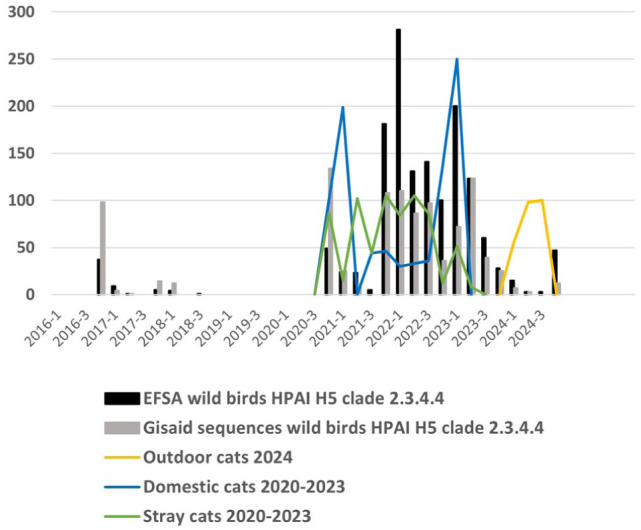
Sampling period of cat cohorts (2020–2024) compared with the presence of highly pathogenic avian influenza (HPAI) H5 clade 2.3.4.4-positive wild birds in the Netherlands (2016–2024). The *x*-axis depicts the years (in quarters). The *y*-axis depicts the number of cats sampled and the number of HPAI H5 clade 2.3.4.4-positive wild birds, as reported to the European Food Safety Authority (EFSA) and Global Initiative on Sharing All Influenza Data (GISAID)

HPAI H5 virus exposure is obviously also affected by the presence of HPAI H5 virus-positive wild birds in the Netherlands, which may depend to some extent on the targeted geographical sampling (at areas where previously HPAI H5 seropositive stray cats had been detected^11^) as well as the sampling periods. Limited numbers of HPAI H5 virus-infected birds were reported in the Netherlands in 2024 compared with 2020–2023.^4^ To compare the presence of HPAI H5 virus-positive wild birds in the Netherlands during the sampling periods of the cohorts shown in Table 3 (this study and Duijvestijn et al^11^), we graphed the number of HPAI H5-positive wild birds, based on the number of sequences reported to EFSA and GISAID (EpiFLu database), in a timeline together with the number of samples obtained (Figure 4). Domestic cats and stray cats in the previous study^11^ were sampled when large numbers of HPAI H5-infected wild birds were reported in the Netherlands. In contrast, the outdoor cat samples in this study were collected when the number of reports of HPAI H5-infected birds were relatively low. The lower ELISA OD ratios in outdoor cat sera (sampled in 2024) compared with stray cat sera (sampled in 2020–2023) (Figure 3), appears in agreement herewith. The low antibody levels in outdoor cats in this study may be due to the waning of antibodies, which in these generally older (mean age 10.7 years) outdoor cats were induced after exposure in previous years. Studies on IAV antibody longevity in cats – to substantiate this hypothesis – have not been conducted; however, cats are known to produce long-lasting detectable antibodies after natural infection with, or vaccination against, different pathogens.^50^ However, we cannot exclude that other differences (eg, in exposure) contribute to the different antibody levels between outdoor cats and stray cats.

In addition to HPAI H5 exposure, we observed seropositivity in domestic cats with and without known outdoor access in this study and in our previous study^11^ to H1N1_pdm09_, which remained at a constant high level (5.5% and 4.6%, respectively) (Table 3) over the years. This indicates frequent and continuous exposure of these cats to H1N1_pdm09_, presumably through human caretakers.^28^ Seropositivity to both HPAI H5 and H1 was detected in two outdoor cat sera, but it remains unclear if these cats had dual or successive exposure, as the exposure moment cannot be extrapolated based on the presence of antibodies. Dual H1N1_pdm09_ and HPAI H5 virus exposure in the Netherlands is theoretically possible, given the temporal overlap in seasonal H1N1_pdm09_ virus circulation in humans – peaking from mid-November to mid-April, and a similar peak in HPAI H5 virus circulation in wild birds.^51^ If cats are dually infected with different IAVs within the same cell, they may potentially act as a mixing vessel. Reassortants of HPAI H5 and H1 have so far not been detected. However, other reassortant viruses have been detected in cats, including a HPAI H5N6 virus containing genes from H5N6, H9N2 and H7N9 subtypes,^38^ as well as a triple reassortant H3N8 IAV, which was also found in a child from the same household.^39^

## Conclusions

The results of our study emphasise the need to closely monitor exposure to avian and human IAVs in domestic cats with outdoor access, especially in areas with high numbers of birds infected with IAVs, including but not limited to HPAI H5.

## References

[1] Caliendo V Kleyheeg E Beerens N , et al. Effect of 2020–21 and 2021–22 highly pathogenic avian influenza H5 epidemics on wild birds, the Netherlands. Emerg Infect Dis 2024; 30: 50–57. 38040665 10.3201/eid3001.230970 PMC10756359

[bibr2-1098612X261461681] Nederlandse Voedsel-en Warenautoriteit. Kaart met vogelgriep besmette dode wilde vogels – Vogelgriep preventie en bestrijding. https://www.nvwa.nl/onderwerpen/dier/vogelgriep/wat-is#anker-5-waar-komt-vogelgriep-voor (2021, accessed 17 June 2021).

[bibr3-1098612X261461681] Reinartz R Slaterus R Foppen R , et al. Update of the target list of wild bird species for passive surveillance of H5 HPAI viruses in the EU. EFSA Support Publ 2024; 21: 8807E.

[4] Sovon. Monitoring dode vogels. https://portal.sovon.nl/dood/result (2024, accessed 24 December 2024).

[5] European Food Safety Authority, European Centre for Disease Prevention and Control, European Union Reference Laboratory for Avian Influenza; Fusaro A , et al. Avian influenza overview December 2023–March 2024. EFSA J 2024; 22. DOI: 10.2903/j.efsa.2024.8754. 10.2903/j.efsa.2024.8754 PMC10977096 38550271

[bibr6-1098612X261461681] Chestakova IV van der Linden A Bellido Martin B , et al. High number of HPAI H5 virus infections and antibodies in wild carnivores in the Netherlands, 2020–2022. Emerg Microbes Infect 2023; 12. DOI: 10.1080/22221751.2023.2270068. 10.1080/22221751.2023.2270068 PMC10732216 37842795

[7] ENETWILD Consortium Flavia O Sascha K , et al. The role of mammals in avian influenza: a review. EFSA Support Publ 2024; 21: 8692E.

[8] Briand F-X Souchaud F Pierre I , et al. Highly pathogenic avian influenza A(H5N1) clade 2.3.4.4b virus in domestic cat, France, 2022. Emerg Infect Dis 2023; 29: 1696–1698. 37379514 10.3201/eid2908.230188 PMC10370847

[bibr9-1098612X261461681] Chothe SK Srinivas S Misra S , et al. Marked neurotropism and potential adaptation of H5N1 clade 2.3.4.4.b virus in naturally infected domestic cats. Emerg Microbes Infect 2024; 14. DOI: 10.1080/22221751.2024.2440498. 10.1080/22221751.2024.2440498 PMC11654043 39648950

[bibr10-1098612X261461681] Domańska-Blicharz K Świȩtoń E Świa̧talska A , et al. Outbreak of highly pathogenic avian influenza A(H5N1) clade 2.3.4.4b virus in cats, Poland, June to July 2023. Euro Surveill 2023; 28. DOI: 10.2807/1560-7917.ES.2023.28.31.2300366. 10.2807/1560-7917.ES.2023.28.31.2300366 PMC10401911 37535474

[bibr11-1098612X261461681] Duijvestijn MBHM Schuurman NNMP Vernooij JCM , et al. Highly pathogenic avian influenza (HPAI) H5 virus exposure in domestic cats and rural stray cats, the Netherlands, October 2020 to June 2023. Euro Surveill 2024; 29. DOI: 10.2807/1560-7917.ES.2024.29.44.2400326. 10.2807/1560-7917.ES.2024.29.44.2400326 PMC11528901 39484684

[bibr12-1098612X261461681] Kang Y-M Heo G-B An S-H , et al. Highly pathogenic avian influenza A(H5N1) virus infection in cats, South Korea, 2023. Emerg Infect Dis 2024; 30: 2510–2520. 39499954 10.3201/eid3012.240154 PMC11616667

[bibr13-1098612X261461681] Sillman SJ Drozd M Loy D , et al. Naturally occurring highly pathogenic avian influenza virus H5N1 clade 2.3.4.4b infection in three domestic cats in North America during 2023. J Comp Pathol 2023; 205: 17–23. 37586267 10.1016/j.jcpa.2023.07.001

[14] Bessière P Brun J Hayes B , et al. Cats as sentinels of mammal exposure to H5Nx avian influenza viruses: a seroprevalence study, France, December 2023 to January 2025. Euro Surveill 2025; 30. DOI: 10.2807/1560-7917.ES.2025.30.12.2500189. 10.2807/1560-7917.ES.2025.30.12.2500189 PMC11951414 40156347

[15] Yamamoto Y Nakamura K Yamada M , et al. Persistence of avian influenza virus (H5N1) in feathers detached from bodies of infected domestic ducks. Appl Environ Microbiol 2010; 76: 5496–5499. 20581177 10.1128/AEM.00563-10 PMC2918962

[16] Leschnik M Weikel J Möstl K , et al. Subclinical infection with avian influenza A(H5N1) virus in cats. Emerg Infect Dis 2007; 13: 243–247. 17479886 10.3201/eid1302.060608 PMC2725870

[bibr17-1098612X261461681] Klopfleisch R Wolf PU Uhl W , et al. Distribution of lesions and antigen of highly pathogenic avian influenza virus A/Swan/Germany/R65/06 (H5N1) in domestic cats after presumptive infection by wild birds. Vet Pathol 2007; 44: 261–268. 17491066 10.1354/vp.44-3-261

[bibr18-1098612X261461681] Moreno A Bonfante F Bortolami A , et al. Asymptomatic infection with clade 2.3.4.4b highly pathogenic avian influenza A(H5N1) in carnivore pets, Italy, April 2023. Euro Surveill 2023; 28. DOI: 10.2807/1560-7917.ES.2023.28.35.2300441. 10.2807/1560-7917.ES.2023.28.35.2300441 PMC10472752 37650905

[19] Federaal Agentschap voor de veiligheid van de voedselketen, Sciensano, FOD Volksgezondheid and Veiligheid van de Voedselketen en Leefmilieu. Gezamenlijk persbericht van het FAVV, Sciensano en de FOD Volksgezondheid, Veiligheid van de Voedselketen en Leefmilieu | Federaal Agentschap voor de veiligheid van de voedselketen. https://favv-afsca.be/nl/publication/gezamenlijk-persbericht-van-het-favv-sciensano-en-de-fod-volksgezondheid-veiligheid-van-de (2000, accessed 5 March 2025).

[20] Rabalski L Milewska A Pohlmann A , et al. Emergence and potential transmission route of avian influenza A(H5N1) virus in domestic cats in Poland, June 2023. Euro Surveill 2023; 28. DOI: 10.2807/1560-7917.ES.2023.28.31.2300390. 10.2807/1560-7917.ES.2023.28.31.2300390 PMC10401914 37535471

[21] Lee K Yeom M Hang Vu TT , et al. Characterization of highly pathogenic avian influenza A (H5N1) viruses isolated from cats in South Korea, 2023. Emerg Microb Infect 2023; 13. DOI: 10.1080/22221751.2023.2290835. 10.1080/22221751.2023.2290835 PMC10810616 38044871

[22] Burrough ER Magstadt DR Petersen B , et al. Highly pathogenic avian influenza A(H5N1) clade 2.3.4.4b virus infection in domestic dairy cattle and cats, United States, 2024. Emerg Infect Dis 2024; 30: 1335–1343. 38683888 10.3201/eid3007.240508 PMC11210653

[23] Acevedo HD Beeler E Crossley B , et al. Salpingitis and multiorgan lesions caused by highly pathogenic avian influenza A(H5N1) virus in a cat associated with consumption of recalled raw milk in California. J Vet Diagn Invest. Epub ahead of print 29 January 2026. DOI: 10.1177/10406387251413563. PMC12858390 41612773

[24] U.S. Department of Agriculture, Animal and Plant Health Inspection Service. HPAI detections in mammals. https://www.aphis.usda.gov/livestock-poultry-disease/avian/avian-influenza/hpai-detections/mammals (1997, accessed 16 January 2025).

[25] Donlevy K. Brooklyn kitten contracted avian flu from another infected cat. New York Post. https://nypost.com/2025/03/15/us-news/brooklyn-kitten-contracted-avian-flu-from-another-infected-cat/ (2025, accessed 19 March 2025).

[26] Vaughan A Joyce A Traub E , et al. Serologic evidence of highly pathogenic avian influenza A(H5N1) virus infection in a veterinary professional exposed to an infected domestic cat – Los Angeles County, California, December 2024–January 2025. MMWR Morb Mortal Wkly Rep 2026; 75: 215–220. 42096344 10.15585/mmwr.mm7517a1 PMC13152193

[27] Naraharisetti R. Highly pathogenic avian influenza A(H5N1) virus infection of indoor domestic cats within dairy industry worker households – Michigan, May 2024. MMWR Morb Mortal Wkly Rep 2025; 74: 61–65. 39977383 10.15585/mmwr.mm7405a2 PMC12370256

[28] Fiorentini L Taddei R Moreno A , et al. Influenza A pandemic (H1N1) 2009 virus outbreak in a cat colony in Italy. Zoonoses Public Health 2011; 58: 573–581. 21824359 10.1111/j.1863-2378.2011.01406.x

[bibr29-1098612X261461681] Löhr CV DeBess EE Baker RJ , et al. Pathology and viral antigen distribution of lethal pneumonia in domestic cats due to pandemic (H1N1) 2009 influenza A virus. Vet Pathol 2010; 47: 378–386. 20382823 10.1177/0300985810368393 PMC4165647

[bibr30-1098612X261461681] Umar S Kim S Gao D , et al. Evidence of reverse zoonotic transmission of human seasonal influenza A virus (H1N1, H3N2) among cats. Influenza Other Respir Viruses 2024; 18. DOI: 10.1111/irv.13296. 10.1111/irv.13296 PMC11026177 38637721

[bibr31-1098612X261461681] van den Brand JM Stittelaar KJ van Amerongen G , et al. Experimental pandemic (H1N1) 2009 virus infection of cats. Emerg Infect Dis 2010; 16: 1745–1747. 21029533 10.3201/eid1611.100845 PMC3294532

[32] Zhang X Shen Y Du L , et al. Serological survey of canine H3N2, pandemic H1N1/09, and human seasonal H3N2 influenza viruses in cats in northern China, 2010–2014. Virol J 2015; 12. DOI: 10.1186/s12985-015-0285-5. 10.1186/s12985-015-0285-5 PMC4391672 25889762

[33] Kuiken T Rimmelzwaan G van Riel D , et al. Avian H5N1 influenza in cats. Science 2004; 306. DOI: 10.1126/science.1102287. 10.1126/science.1102287 15345779

[bibr34-1098612X261461681] Reperant L van de Bildt MW van Amerongen G , et al. Marked endotheliotropism of highly pathogenic avian influenza virus H5N1 following intestinal inoculation in cats. J Virol 2012; 86: 1158–1165. 22090101 10.1128/JVI.06375-11 PMC3255817

[bibr35-1098612X261461681] Rimmelzwaan GF van Riel D Baars M , et al. Influenza A virus (H5N1) infection in cats causes systemic disease with potential novel routes of virus spread within and between hosts. Am J Pathol 2006; 168: 176–183; quiz 364. 16400021 10.2353/ajpath.2006.050466 PMC1592682

[36] Vahlenkamp TW Teifke JP Harder TC , et al. Systemic influenza virus H5N1 infection in cats after gastrointestinal exposure. Influenza Other Respir Viruses 2010; 4: 379–386. 20958932 10.1111/j.1750-2659.2010.00173.x PMC4634607

[37] Zhao S Schuurman N Tieke M , et al. Serological screening of influenza A virus antibodies in cats and dogs indicates frequent infection with different subtypes. J Clin Microbiol 2020; 58. DOI: 10.1128/JCM.01689-20. 10.1128/JCM.01689-20 PMC7587082 32878956

[38] Cao X Yang F Wu H , et al. Genetic characterization of novel reassortant H5N6-subtype influenza viruses isolated from cats in eastern China. Arch Virol 2017; 162: 3501–3505. 28730524 10.1007/s00705-017-3490-2

[bibr39-1098612X261461681] Bao P Liu Y Zhang X , et al. Human infection with a reassortment avian influenza A H3N8 virus: an epidemiological investigation study. Nat Commun 2022; 13. DOI: 10.1038/s41467-022-34601-1. 10.1038/s41467-022-34601-1 PMC9649012 36357398

[40] Zhao J He W Lu M , et al. Emergence and characterization of a novel reassortant canine influenza virus isolated from cats. Pathogens 2021; 10. DOI: 10.3390/pathogens10101320. 10.3390/pathogens10101320 PMC8539923 34684269

[41] Duijvestijn MBHM Schuurman NNMP Vernooij JCM , et al. Serological survey of retrovirus and coronavirus infections, including SARS-CoV-2, in rural stray cats in the Netherlands, 2020–2022. Viruses 2023; 15. DOI: 10.3390/v15071531. 10.3390/v15071531 PMC10385588 37515217

[42] Arnold ME Slomka MJ Breed AC , et al. Evaluation of ELISA and haemagglutination inhibition as screening tests in serosurveillance for H5/H7 avian influenza in commercial chicken flocks. Epidemiol Infect 2018; 146: 306–313. 29325601 10.1017/S0950268817002898 PMC9134519

[43] Jensen TH Ajjouri G Handberg KJ , et al. An enzyme-linked immunosorbent assay for detection of avian influenza virus subtypes H5 and H7 antibodies. Acta Vet Scand 2013; 55. DOI: 10.1186/1751-0147-55-84. 10.1186/1751-0147-55-84 PMC4176992 24256721

[44] Shu Y McCauley J. GISAID: Global initiative on sharing all influenza data – from vision to reality. Euro Surveill 2017; 22. DOI: 10.2807/1560-7917.ES.2017.22.13.30494. 10.2807/1560-7917.ES.2017.22.13.30494 PMC5388101 28382917

[45] Cecchetti M Crowley SL Goodwin CED , et al. Contributions of wild and provisioned foods to the diets of domestic cats that depredate wild animals. Ecosphere 2021; 12. DOI: 10.1002/ecs2.3737.

[46] Ende MVD Strijkstra A Dias E , et al. Spatial ecology and prey choice of tagged feral cats on the island of Schiermonnikoog. Lutra 2017; 60: 73–91.

[47] Yamamoto Y Nakamura K Mase M. Survival of highly pathogenic avian influenza H5N1 virus in tissues derived from experimentally infected chickens. Appl Environ Microbiol 2017; 83. DOI: 10.1128/AEM.00604-17. 10.1128/AEM.00604-17 PMC5541213 28625993

[48] Castañeda I Forin-Wiart M-A Pisanu B , et al. Spatiotemporal and individual patterns of domestic cat (*Felis catus*) hunting behaviour in France. Animals (Basel) 2023; 13. DOI: 10.3390/ani13223507. 10.3390/ani13223507 PMC10668736 38003125

[49] Bouma EMC Reijgwart ML Dijkstra A. Family member, best friend, child or ‘just’ a pet, owners’ relationship perceptions and consequences for their cats. Int J Environ Res Public Health 2022; 19. DOI: 10.3390/ijerph19010193. 10.3390/ijerph19010193 PMC8750854 35010452

[50] Schultz RD Thiel B Mukhtar E , et al. Age and long-term protective immunity in dogs and cats. J Comp Pathol 2010; 142 Suppl 1: S102–S108. 10.1016/j.jcpa.2009.10.009 19959181

[51] Rijksinstituut voor Volksgezondheid en Milieu. Feiten en cijfers griep. https://www.rivm.nl/griep-griepprik/feiten-en-cijfers (1990, accessed 5 April 2024).

